# Genotyping of virulent *Escherichia coli* obtained from poultry and poultry farm workers using enterobacterial repetitive intergenic consensus-polymerase chain reaction

**DOI:** 10.14202/vetworld.2017.1292-1296

**Published:** 2017-11-01

**Authors:** M. Soma Sekhar, N. Mohammad Sharif, T. Srinivasa Rao, M. Metta

**Affiliations:** 1 Department of Veterinary Public Health and Epidemiology, NTR College of Veterinary Science, Sri Venkateswara Veterinary University, Gannavaram, Andhra Pradesh, India; 2 Department of Veterinary Microbiology, College of Veterinary Science, Tirupati, Andhra Pradesh, India; 3 Department of Animal Genetics and Breeding, NTR College of Veterinary Science, Gannavaram, Andhra Pradesh, India

**Keywords:** *Escherichia coli*, enterobacterial repetitive intergenic consensus-polymerase chain reaction, genotype, poultry, serotype, virulent

## Abstract

**Aim:**

The aim of this study was to characterize virulent *Escherichia coli* isolated from different poultry species and poultry farm workers using enterobacterial repetitive intergenic consensus-polymerase chain reaction (ERIC-PCR) genotyping.

**Materials and Methods:**

Fecal swabs from different poultry species (n=150) and poultry farm workers (n=15) were analyzed for *E. coli* and screened for virulence genes (*stx1*, *stx2, eaeA*, and *hlyA*) by multiplex PCR. Virulent *E. coli* was serotyped based on their “O” antigen and then genotyped using ERIC-PCR.

**Results:**

A total of 134 *E. coli* isolates (122/150 from poultry and 12/15 from farm workers) were recovered. Virulence genes were detected in a total of 12 isolates. Serological typing of the 12 virulent *E. coli* revealed nine different serotypes (O2, O49, O60, O63, O83, O101, O120, UT, and Rough). ERIC-PCR genotyping allowed discrimination of 12 virulent *E. coli* isolates into 11 ERIC-PCR genotypes. The numerical index of discrimination was 0.999.

**Conclusion:**

Our findings provide information about the wide genetic diversity and discrimination of virulent *E. coli* in apparently healthy poultry and poultry farm workers of Andhra Pradesh (India) based on their genotype.

## Introduction

Shiga toxigenic *Escherichia coli* (STEC) is known to cause a range of human foodborne illnesses such as hemorrhagic colitis (HC), hemolytic uremic syndrome (HUS), and thrombotic thrombocytopenic purpura (TTP) [1]. The pathogenicity of STEC is mainly mediated by virulence genes such as *stx1* (Shiga toxin type-1), *stx2* (Shiga toxin type-2), *eaeA* (encodes intimin, which facilitates intimate adherence of bacteria to intestinal epithelial cells, resulting in the production of attaching and effacing lesion), and *hlyA* (enterohemolysin) [2]. Foodborne disease outbreaks, HUS, TTP, and HC are associated with certain STEC O-serogroups mainly including O157, O26, O91, O103, O121, O113, O111, O145, O45, and O128 as well as untypeable groups [3-5]. Compared to cattle and sheep, which act as major reservoirs of STEC, a limited number of reports have been published on STEC from poultry species and their genetic relatedness with human STEC [6-8].

Bacterial genomes contain imperfect palindromic repeat sequences such as enterobacterial repetitive intergenic consensus (ERIC) sequence [9]. The distance between these repetitive sequences and frequency of repeated sequences vary with individual bacterial strains [10]. ERIC-PCR genotyping technique yields strain-specific unique multiband patterns obtained by the amplification of conserved ERIC sequences, thus discriminating individual bacterial strains [11]. ERIC-PCR technique can be effectively used to discriminate *E. coli* strains intra-serotypically into different genotypes [10].

Perusal of the available literature revealed the paucity of information regarding the intra-serotypic genetic diversity of virulent *E. coli* of poultry origin in India. Hence, the present research was undertaken to assess the genetic diversity of virulent *E. coli* isolated from poultry and poultry farm workers in Andhra Pradesh, India.

## Materials and Methods

### Ethical approval

Ethical approval is not necessary to pursue this study. However, samples were collected without harming birds. Informed consent was obtained from farm workers.

### Sample collection and isolation of *E. coli*

Fecal swabs (n=150) from different poultry species, namely, chicken, ducks, quails, turkey, fancy birds (each 30), and poultry farm workers (n=15) were collected from three different poultry farms in Andhra Pradesh, India. Isolation and identification were done using Trypticase Soy Broth (Hi-Media), eosin methylene blue (EMB, HiMedia) agar, and MacConkey agar medium. The isolates were confirmed to be *E. coli* by standard biochemical tests [12] as well as by the PCR amplification of the *E16S* gene of *E. coli* using the primer pair (F, 5’- ATC AAC CGA GAT TCC CCC AGT-3’ and R, 5’- TCA CTA TCG GTC AGT CAG GAG-3’) [13]. Whole-cell DNA extraction was carried out by boiling and snap chilling method [14].

### Detection of virulence genes by multiplex PCR

A multiplex (m)-PCR for the detection of *stx*_1_, *stx*_2_, *eaeA*, and *hlyA* genes in *E. coli* was carried out as described by Paton and Paton [1]. Primers used and their expected amplicon sizes are summarized in Table-1. The reaction was carried out in 10 µl volume containing 5.0 µl of PCR Master Mix (Takara Bio Inc., Japan), 0.22 µl of each forward and reverse primer (10.0 pmol/µl), 2.24 µl of nuclease-free water, and 1.0 µl of DNA template (75 ng/µl). Thermal cycling conditions were as follows - 35 cycles, each consisting denaturation at 95°C for 60 s, annealing at 65°C for 120 s, and elongation at 72°C for 90 s (for first 10 cycles), decrementing annealing temperature to 60°C by cycle 15; denaturation at 95°C for 60 s, annealing at 60°C 120 s, and elongation at 72°C 90 s (from cycle 15-25), and incrementing elongation time from 90 s to 150 s from cycles 26 to 35.

**Table-1:** Primers used for detection of virulence genes in *E. coli*.

Primer	Primer sequence (5’-3’)	Target gene name	Amplicon size	Reference
*Stx 1*	F: ATAAATCGCCATTCGTTGACTAC R: AGAACGCCCACTGAGATCATC	Shiga toxin-1	180	Paton and Paton (1998)
*Stx 2*	F: GGCACTGTCTGAAACTGCTCC R: TCGCCAGTTATCTGACATTCTG	Shiga toxin-2	255	
*eaeA*	F: GACCCGGCACAAGCATAAGC R: CCACCTGCAGCAACAAGAGG	Intimin	384	
*hlyA*	F: GCATCATCAAGCGTACGTTCC R: AATGAGCCAAGCTGGTTAAGCT	Hemolysin	534	

*E. coli=Escherichia coli*.

### Serotyping of virulent *E. coli* isolates

Serotyping of *E. coli* isolates positive for virulence genes was done at National *Salmonella* and *Escherichia coli* Center, Central Research Institute, Kasauli (Himachal Pradesh, India) on the basis of their “O” antigen.

### ERIC-PCR genotyping of virulent *E. coli* isolates

Genotyping of virulent *E. coli* isolates was done using oligonucleotide primers, ERIC-1 (5’-ATG TAA GCT CCT GGG GAT TCA C-3’) and ERIC-2 (5’-AAG TAA GTG ACT GGG GTG AGC G-3’) [15]. Reaction was carried out in 25 µl volume using 12 μl of Master Mix (Takara Bio Inc.), 1.5 µl of DNA template (100 ng/μl), 1.0 μl of each primer (20 pmol/μl), and 9.5 μl of nuclease-free water. The DNA amplifications were performed in the Eppendorf thermal cycler (USA) with an initial denaturation at 95°C for 7 min, followed by 30 cycles of 30 s of denaturation at 94°C, 1 min annealing at 50°C, 8 min of elongation at 65°C, and 16 min of final elongation at 65°C. Standardization of the ERIC-PCR reactions was done using the DNA from reference strain *E. coli* (MTCC 1610).

### Analysis of DNA fingerprinting patterns

The PCR products were resolved by 2% agarose gel electrophoresis under 110V for 2 h [16]. Banding patterns were photographed using BIO-RAD Gel Documentation system (USA). The DNA fingerprints obtained were analyzed both by visual inspection and Image Lab Software (BIO-RAD). The position of bands was compared using 100 bp and 1 kb DNA ladder (Genei^™^, Bengaluru). Binary matrix was constructed based on the presence (1) or absence (0) of a particular band in the given strain. Dendrogram was constructed by “branch-and-bound method” using dollop program of PHYLIP software, version 3.6. Clusters were considered at a 70% similarity cutoff value. The similarity of banding patterns was determined using the Pearson’s correlation coefficient. The numerical index of discrimination was calculated using Simpson’s index of diversity, D = 1-1/N (N-1) ∑ n_j_ (n_j_-1)_,_ where D corresponds to the discriminatory power, N corresponds to the total number of strains, and n_j_ corresponds to the number of strains belonging to the j^th^ type [17].

## Results and Discussion

### Isolation and identification of *E. coli*

Of the 150 poultry fecal swab samples analyzed, a total of 122 (81.3%) *E. coli* isolates were recovered (26/30 from chicken, 24/30 ducks, 23/30 quails, 24/30 turkey, and 25/30 fancy birds). Of the 15 human fecal swab samples analyzed, a total of 12 (80%) *E. coli* were recovered. All the *E. coli* isolates were able to produce green metallic-sheen colonies on EMB agar, lactose-fermenting pink colonies on MacConkey agar, and amplified *E16S* gene giving 231 bp amplicon. The present results were in accordance with the findings of earlier studies from Srinagar, India [18] and Sao Paulo, Brazil [8], where *E. coli* was isolated from 80.2% and 78.3% of the poultry fecal samples tested.

### Virulence genes characterization

The multiplex-PCR assay revealed the presence of one or more virulence genes in a total of 12 (chicken, 3; ducks, 2; turkey, 2; fancy birds, 2; quails, 1; and humans, 2) isolates, with an overall frequency of 12% (10/122 *E. coli*) and 13% (2/15 *E. coli*) occurrence of virulence genes in poultry and poultry farm workers, respectively. All these 12 isolates were positive for both *stx1* and *eaeA* genes, whereas *hlyA* gene was detected in eight isolates and *stx2* gene in four isolates (Table-2). The previous studies on avian pathogenic *E. coli* by Parreira and Gyles [19] and Dutta *et al*. [7] revealed association of STEC with 53% and 23.8% of avian pathogenic *E. coli* isolates. However, the absence of STEC is also reported in another study where *E. coli* was recovered from apparently healthy birds [20]. In the present study, all the 12 *E. coli* isolates that were positive for at least one virulence gene carried both *stx1* and *eaeA* genes, which is in agreement with the findings of other workers [7,19,20], who reported a higher percentage (>50%) of pathogenic *E. coli* isolates carrying *stx1* and *eaeA* genes. However, in contrast to our study, Wani *et al*. [18] reported only 2.4% frequency of *eaeA* gene in *E. coli* from poultry.

**Table-2:** Serotypes and ERIC-PCR genotypes of virulent *E. coli*.

Number of samples tested	Number positive for *E. coli*	*E. coli* positive for virulence genes	Number of *E. coli* isolates carrying particular virulence gene				Serotypes detected in the virulent *E. coli*	Number of ERIC genotypes detected

			*stx1*	*stx2*	*eaeA*	*hlyA*		
Chicken (30)	26	3	3	2	3	2	O2, O120	3 (E1, E2, E3)
Ducks (30)	24	2	2	-	2	1	O63, R	2 (E4, E5)
Quails (30)	23	1	1	-	1	-	UT	1 (E6)
Turkey (30)	24	2	2	-	2	2	O83, UT	2 (E7, E8)
Fancy birds (30)	25	2	2	2	2	2	O60	1 (E9, E9)
Humans (15)	12	2	2	-	2	1	O49, O101	2 (E10, E11)
Total (165)	134	12	12	4	12	8	9	11

*E. coli=Escherichia coli,* ERIC=Enterobacterial repetitive intergenic consensus, PCR=Polymerase chain reaction

### Serological typing

Serotyping of 12 virulent *E. coli* isolates revealed nine different serotypes, of which O2, O60 and UT were predominant (two isolates each), followed by O49, O63, O83, O101, O120, and Rough (one isolate each). Detection of O2, O60, O120, and R serotypes among *E. coli* of avian origin in the present study was in accordance with a study from Jammu and Kashmir [18]. However, avian *E. coli* serotypes reported in other studies from India [6,7] were not detected in the present study, indicating the variable distribution of different serotypes of *E. coli* in different geographical regions of India.

### ERIC-PCR genotypes among virulent *E. coli*

**Figure-1:**
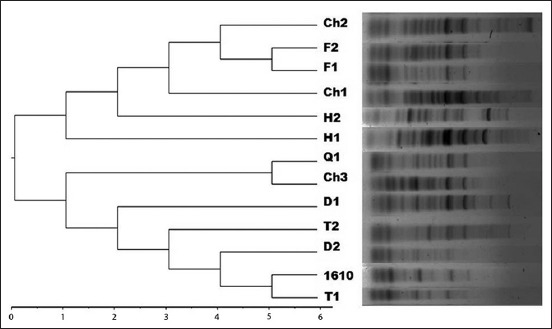
Enterobacterial repetitive intergenic consensus-polymerase chain reaction cluster analysis of fecal *Escherichia coli* isolates from chicken (Ch1, Ch2, and Ch3), ducks (D1 and D2), quails (Q1), turkey (T1 and T2), fancy birds (F1 and F2), and poultry farm workers (H1 and H2); standard *E. coli* MTCC 1610.

ERIC-PCR genotyping revealed 7-18 fragments resolved per isolate, ranging in size from slightly less than 100 bp to 2000 bp (Figure-1). The binary data of ERIC-PCR profiles showed highly polymorphic DNA fragments among the 12 virulent *E. coli* isolates and reference strain of *E. coli*, namely, 7, 8, 9, 12, 13, 14, 15, 17, and 20 amplicons detected in 1, 2, 1, 2, 2, 1, 1, 1, and 2 isolates, respectively. ERIC-PCR profiles revealed 11 ERIC-PCR genotypes discriminated among 12 *E. coli* isolates. The two virulent *E. coli* isolates (F1 and F2) recovered from fancy birds displayed a single ERIC-PCR fingerprinting profile (E9) (Figure-1). Interestingly, these two isolates shared same “O” serotype (O60) as well. The present observations were in accordance with the findings of Prabhu *et al*. [10], who assessed the utility of ERIC-PCR technique for the intra-serotypic differentiation of *E. coli* strains isolated from episodes of bovine mastitis.

Several investigations have used ERIC-PCR as a highly sensitive genomic fingerprinting tool for detecting and determining different strains of *E. coli* from diverse sources [11,15,21]. Soltani *et al*. recognized 65 different avian *E. coli* strains from 95 samples with 232 to 2690 bp bands on gel electrophoresis and confirmed that ERIC-PCR as a good technique for bacterial genotyping among cheap and simple molecular tools [22]. In determining genetic profiles of *E. coli*, ERIC-PCR technique was showed to have more discriminatory power than 16S rRNA gene analysis [23].

In the present study, ERIC-PCR profile dendrogram analysis discriminated virulent *E. coli* isolates into three major clusters (C_1_ to C_3_) for 70% similarity cutoff, namely, C_1_ with four isolates (chicken, 2; fancy birds, 2), C_2_ with two isolates (chicken, 1; quail, 1), and C_3_ with five isolates (turkey, 2; duck, 2; and reference strain *E. coli* MTCC 1610) (Figure-1). The two human *E. coli* isolates were found to be unclustered (UC) with other isolates. Cluster analysis indicated wide intra-serotypic genetic diversity among the isolates affiliated to the same “O” serotype, as evidenced by their grouping under different clusters in the dendrogram. For example, isolates of O120 serotype, namely, Ch2 and Ch3 were grouped under clusters C_1_ and C_2_, respectively, with different ERIC-PCR genotypes (E2 and E3) (Table-2 and Figure-1). The numerical index of discrimination calculated for 12 isolates, using Simpson’s index of diversity was 0.997, which corroborate with earlier studies [15,21], where ERIC-PCR was shown to have highly significant discriminatory power in characterizing the genetic diversity of *E. coli* strains.

## Conclusion

The results presented in this paper and past studies emphasize the utility of the combination of serotyping with PCR-based genotyping tools in the epidemiological investigations of virulent *E. coli* strains. This is the first study in India to elucidate the intra-serotypic genetic diversity of virulent *E. coli* isolates from different poultry species as well as poultry farm workers, and ERIC-PCR was proved to be quick, sharp, and cost-effective fingerprint method for effective discrimination of *E. coli* strains based on their genotype.

## Authors’ Contributions

MSS and NMS prepared the study design and carried out the research under the supervision of TSR and MM. NMS collected the samples and executed the isolation. MSS and MM conducted the molecular part of the research. The manuscript was drafted and revised by MSS and NMS under the guidance of TSR and MM. All authors read and approved the final manuscript.
